# A rare manifestation of a common problem: resting ST-segment depression on the exercise treadmill test: Case report

**DOI:** 10.1097/MD.0000000000041981

**Published:** 2025-05-09

**Authors:** Yan Tan, Dandan Lai, Qifeng Jing

**Affiliations:** a Department of Cardiology, Xiaoshan Affiliated Hospital of Wenzhou Medical University, Hangzhou, Zhejiang, China; b Department of Radiology, Xiaoshan Affiliated Hospital of Wenzhou Medical University, Hangzhou, Zhejiang, China.

**Keywords:** exercise treadmill test, myocardial bridge, ST-segment depression

## Abstract

**Rationale:**

Myocardial bridge (MB), where a coronary artery segment is overlaid by myocardium, is often asymptomatic but can lead to serious complications. This case highlights a rare electrocardiographic manifestation of MB: resting ST-segment depression when transitioning from a supine to a standing position.

**Patient concerns:**

A 39-year-old male with no significant medical history presented with intermittent, nonexertional chest pain. His resting electrocardiogram was normal in the supine position but showed ST-segment depression in leads II, III, aVF, and V5-V6 when standing.

**Diagnoses:**

Coronary angiography confirmed a MB in the left anterior descending artery.

**Interventions:**

The patient was managed with beta-blockers, aspirin, and rosuvastatin therapy.

**Outcomes:**

After 8 months of follow-up, no complications or cardiac symptoms were observed.

**Lessons:**

This case underscores the importance of recognizing positional ST-segment changes as a potential indicator of MB, offering valuable insights into the diagnosis and management of this condition.

## 1. Introduction

A myocardial bridge (MB) refers to the portion of the myocardium that overlies an intracardiac segment of the coronary artery, usually the left anterior descending (LAD) artery. The presentation can range from asymptomatic to presenting as acute coronary syndrome, ventricular tachycardia, or sudden death. Due to risk factors for malignant arrhythmias and sudden cardiac death, it is crucial to identify patients with MB who require urgent and invasive intervention.

## 2. Case report

**Figure 1.**
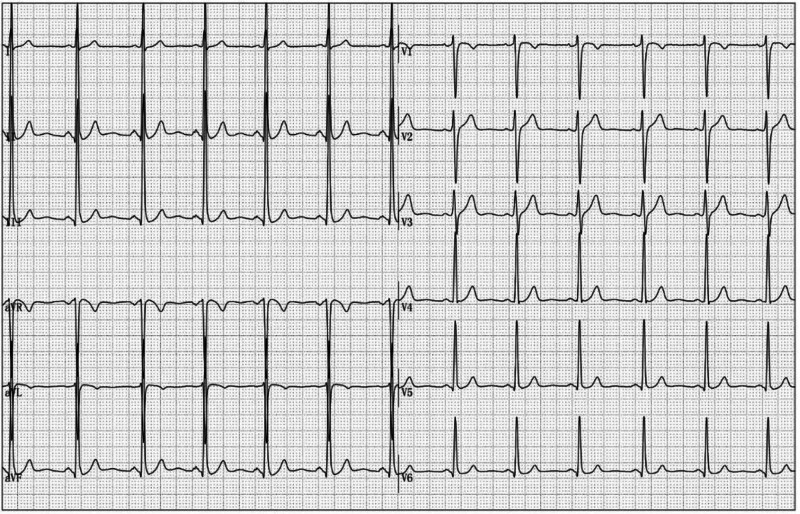
The resting ECG of the patient was normal in the supine position. ECG = electrocardiogram.

**Figure 2.**
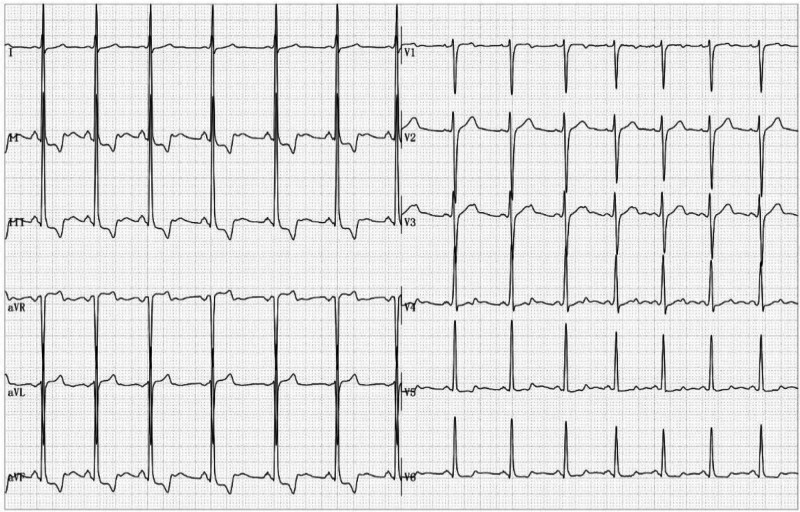
Demonstration of an ST-segment depression in leads II, III, aVF, and V5-V6 in the standing position.

**Figure 3.**
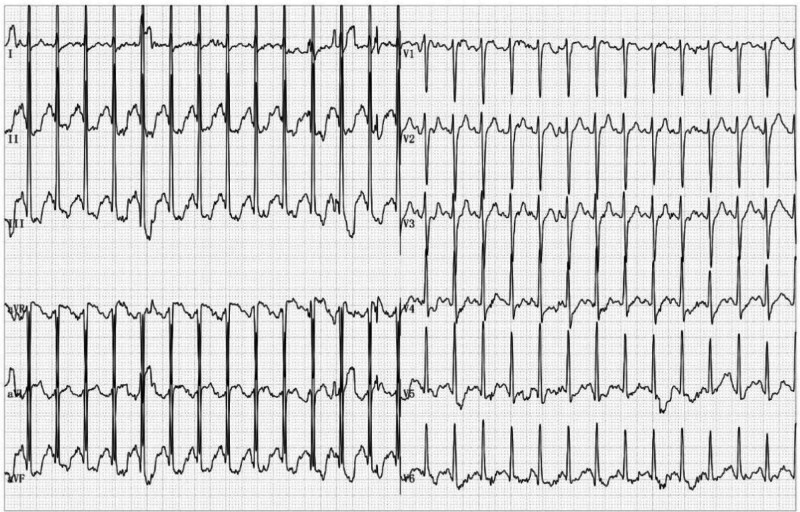
Demonstration of a progressive ST-segment depression in lead II, III, aVF, and V5-V6 presenting inducible myocardial ischemia during the peak of the exercise test.

**Figure 4.**
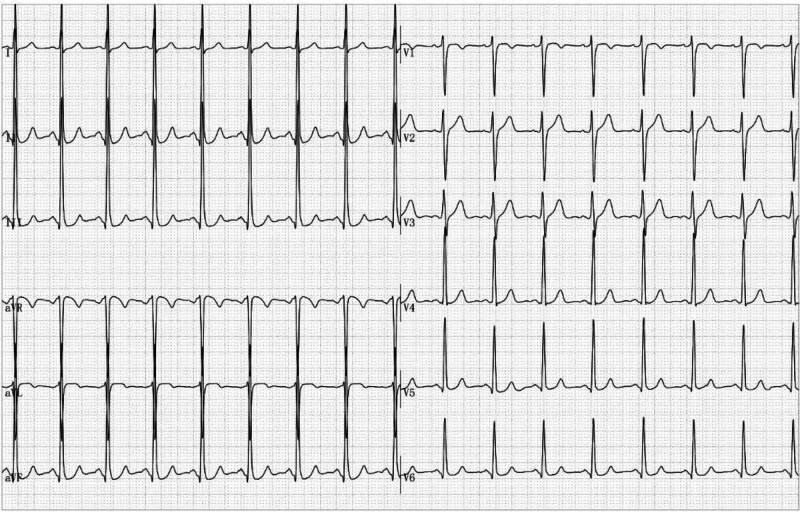
A supine rest ECG showed normal results after 5 min. ECG = electrocardiogram.

A 39-year-old male with no significant medical history was examined by a physician with a complaint of left-sided, intermittent, nonexertional chest pain and pressure for 2 months. Cardiac and systemic examinations were normal; specifically, he had normal heart sounds with no murmurs. The exercise treadmill test (ETT) was organized to exclude exertion-related dysrhythmia. The resting electrocardiogram (ECG) was normal in the supine position but developed an ST-segment depression in leads II, III, aVF, and V5-V6 in the standing position (Figs. 1 and 2). A progressive ST-segment depression manifestation was seen in lead II, III, aVF, and V5-V6 presenting inducible myocardial ischemia at the peak of the exercise test (Fig. 3). A supine rest ECG showed normal results after 5 minutes (Fig. 4). He denied any chest pain or dyspnea during the whole ETT.

**Figure 5.**
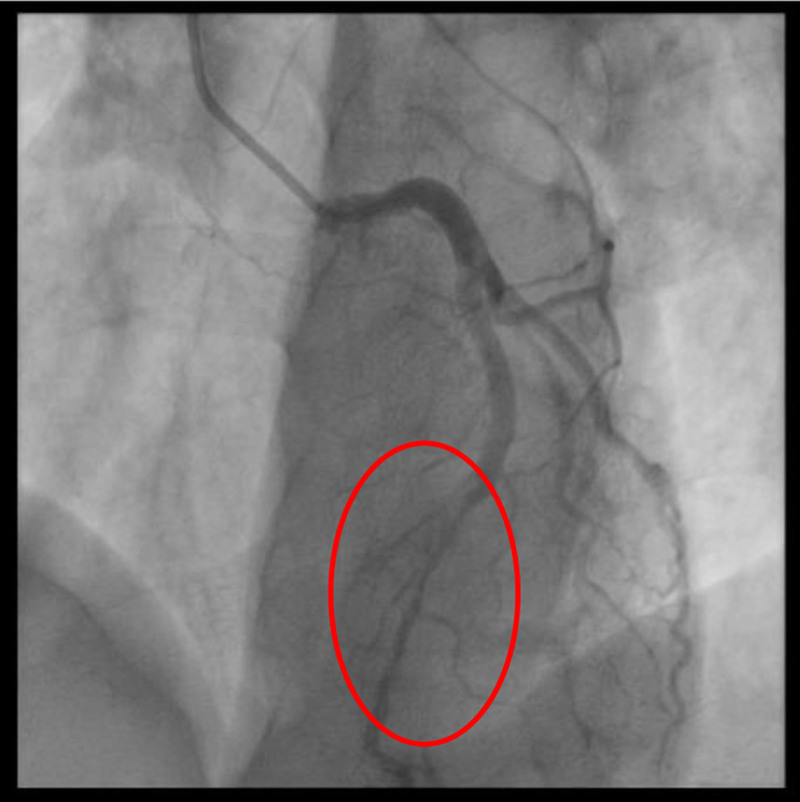
Coronary angiography showed a systolic narrowing of the mid-LAD. LAD = left anterior descending.

**Figure 6.**
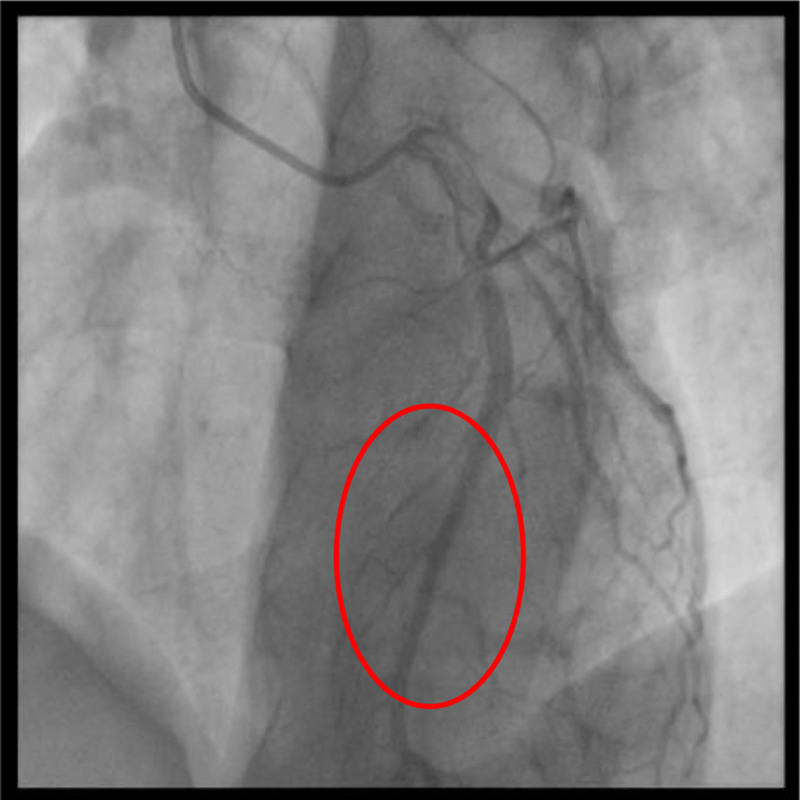
Coronary angiography of left anterior descending artery in diastole.

Subsequently, the patient was referred to our cardiology clinic for further evaluation. The echocardiographic findings were normal. Laboratory values, including high-sensitivity troponin and electrolytes, were within normal limits. Coronary angiography revealed a MB in the left middle descending artery. The diagnosis was confirmed to be a MB (Figs. 5 and 6, and Supplemental Video S1, Supplemental Digital Content, https://links.lww.com/MD/O891). The patient was treated with beta-blockers, aspirin, and rosuvastatin due to his ongoing ischemia on stress testing. At present, 8 months after treatment, the patient is in good condition without any complications and cardiac symptoms.

## 3. Discussion

According to Lee and Chen,^[1]^ Reyaman first described myocardial cover over the epicardial coronary artery as MB during an autopsy in 1737. Most cases of MB are asymptomatic and benign; however, they can cause ischemia, angina, acute coronary syndrome, arrhythmias, and even sudden death in rare instances.^[2–4]^ It is estimated that 5.7% to 33% of patients diagnosed with coronary computed tomography angiography experience myocardial bridging.^[5]^ In most cases, MB occurs in the LAD artery, and to a lesser extent in the circumflex and diagonal arteries. Bridge thickness, length, myocardial fiber orientation, and presence of adipose tissue or loose connective tissue determine physiological effects.^[6]^

MB is not diagnosed or managed by any major cardiovascular society guidelines. Asymptomatic, resting patients rarely show abnormalities on surface ECGs.^[7]^ The ETT is the first examination performed to assess coronary reserve because it is noninvasive, inexpensive, and safe. Patients with myocardial bridging have electrocardiographic abnormalities during ETT in a range between 28% and 68% of cases.^[5]^ Our patient developed an ST-segment depression in lead II, III, aVF/V5-V6) in the standing position and a progressive ST-segment depression in lead II, III, aVF, and V5-V6 at the peak of the ETT, which prompted an angiogram showing LAD bridging. There is a question as to why this patient has been asymptomatic for all of his adult life. As patients age, certain changes in their hearts such as left ventricular hypertrophy, diastolic dysfunction, and atherosclerosis worsen the supply–demand mismatch.

The present case showed that myocardial bridging may be asymptomatic even with 80% systolic narrowing of the LAD. The ETT was the only abnormality our patient experienced at the early stage. To our knowledge, there are very few case reports or studies at present. The study’s outcomes demonstrated the effectiveness of the chosen treatment strategy and provided valuable insights into the management of MB with positional ST-segment depression. After initiating therapy with beta-blockers, aspirin, and rosuvastatin, the patient experienced complete resolution of symptoms and remained asymptomatic throughout the follow-up period. Specifically, the patient reported no further episodes of chest pain or discomfort, and no cardiac complications were observed during the 8 months of follow-up.

The electrocardiographic findings also showed stabilization, as no further ST-segment depression was observed during subsequent evaluations. The patient’s functional status improved, and he was able to resume normal daily activities without any limitations. In addition, the absence of adverse events related to the medications highlighted the safety and tolerability of the treatment regimen in this case.

These outcomes suggest that the combination of beta-blockers, aspirin, and rosuvastatin is a viable treatment approach for managing MB-associated ischemia and positional ST-segment depression. However, the relatively short follow-up period limits the ability to assess the long-term efficacy and durability of this treatment strategy. Nonetheless, the results underscore the importance of early diagnosis and tailored therapeutic interventions in improving patient outcomes in such cases.

This case report has several limitations. First, as a single case study, the findings may not be generalizable to all patients with MB or those presenting with positional ST-segment depression. The patient’s asymptomatic state during the ETT limits the ability to fully explore the relationship between symptoms and ST-segment changes. In addition, the follow-up period of 8 months is relatively short, making it difficult to assess long-term outcomes or the durability of the treatment strategy. The study also lacks a control group to compare the natural history of MB and its response to therapy, which limits the ability to establish a causal relationship between the interventions (beta-blockers, aspirin, and rosuvastatin) and the observed outcomes. Furthermore, the absence of data on lifestyle modifications or other potential cardiovascular risk factors in the patient’s management plan leaves room for further exploration of complementary therapeutic strategies. Finally, the diagnostic approach was limited to coronary angiography, and the selection of this patient for the study may introduce bias, as not all patients with MB may present similarly.

## 4. Conclusion

In conclusion, myocardial bridging is an important diagnosis and should be paid close attention to patients with abnormal resting ETT. To define the natural history of MB, patient selection, and optimal treatment strategies, randomized trials, as well as long-term registry data are required.

## Author contributions

**Investigation:** Yan Tan.

**Writing – review & editing:** Yan Tan.

**Formal analysis:** Dandan Lai.

**Writing – original draft:** Dandan Lai, Qifeng Jing.

**Conceptualization:** Qifeng Jing.

## Supplementary Material


